# New Insights into the Role of INSL-3 in the Development of Cryptorchidism

**DOI:** 10.3390/children10040737

**Published:** 2023-04-17

**Authors:** Alma-Raluca Lăptoiu, Elena-Lia Spoială, Gabriela Dumitrita Stanciu, Elena Hanganu, Vasile Valeriu Lupu, Carmen-Iulia Ciongradi, Cristina Gavrilovici

**Affiliations:** 1 Pediatrics Department, “Grigore T. Popa” University of Medicine and Pharmacy, 700115 Iasi, Romania; 2 Advanced Research and Development Center for Experimental Medicine (CEMEX), “Grigore T. Popa” University of Medicine and Pharmacy, 16 Universitatii Street, 700115 Iasi, Romania; 3 Department of Biomedical Sciences, “Grigore T. Popa” University of Medicine and Pharmacy, 16 Universitatii Street, 700115 Iasi, Romania; 4 Department of Pediatric and Orthopaedic Surgery, “Sfânta Maria” Emergency Children Hospital, 700309 Iași, Romania; 5 2nd Department of Surgery and Ortophedics, “Grigore T. Popa” University of Medicine and Pharmacy, 16 Universitatii Street, 700115 Iasi, Romania

**Keywords:** undescended testis, cryptorchidism, genetics, insulin-like factor 3 (INSL3)

## Abstract

Cryptorchidism, defined as the failure of at least one or both testicles to descend into the scrotal pouches, is the most frequent (1.6–9% at birth, 1/20 males at birth) congenital anomaly encountered in newborn males, resulting in one of the most frequent causes of non-obstructive azoospermia in men. Similar to other congenital malformations, cryptorchidism is thought to be caused by endocrine and genetic factors, combined with maternal and environmental influences. The etiology of cryptorchidism is unknown, as it involves complex mechanisms aiming to control the testicular development and descent from their initial intra-abdominal location in scrotal pouches. The implication of insulin-like 3 (INSL-3) associated with its receptor (LGR8) is critical. Genetic analysis discloses functionally deleterious mutations in INSL3 and GREAT/LGR8 genes. In this literature review, we discuss and analyze the implication of INSL3 and the INSL3/LGR8 mutation in the occurrence of cryptorchidism in both human and animal models.

## 1. Introduction

Cryptorchidism is the clinical condition in which the testes fail to descend [1] into the scrotum, and it is considered one of the most frequent congenital malformations of the genitourinary tract [2], with an incidence of 1.6% to 9.0% in newborn males [3]. Normally, the testicles descend between 7–8 months of gestation, and approximately 30% of premature male babies have undescended testis, unilateral or bilateral, with the incidence decreasing at almost 3.4% in near-term male babies [4]. The pathogenesis of non-syndromic cryptorchidism is still unclear, and probably multifactorial, implying genetic and environmental risk factors [4]. The main factors involved in the testicular descent are hormones (androgens), the genitofemoral nerve, and the most quoted insulin-like hormone 3 (INSL3). INSL3 controls the transabdominal part of testicular descent, playing a role in the gubernacular swelling reaction [5,6,7]. This literature review aims to discuss and analyze the importance of INSL3 and its mutation into cryptorchidism in both human and animal models.

## 2. Materials and Methods

**Figure 1:**
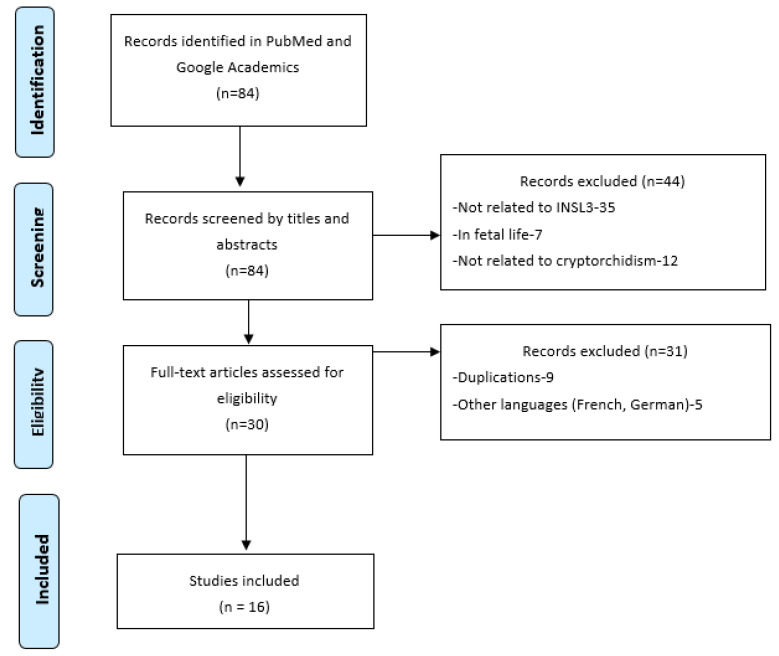
PRISMA flow chart for papers selection.

We conducted a Pubmed and Google Academics search from January 2017 to January 2022, using the following search terms (“INSL3”) OR [(“LGR8”) OR (“INSL-3”)] AND [(“cryptorchidism”) OR (“undescended testis”)]. The literature search retrieved 84 references (see Figure 1). All articles were screened by title and abstracts. We removed those not related to INSL-3 or LGR8. After analyzing 84 full articles, 68 were excluded due to a lack of reference to genetics or other limitations (duplications, other languages, etc.). We added 8 studies after analyzing the references from screened articles. In total, 24 publications were included in this review. All were first manually searched to further identify any additional eligible studies to include.

## 3. Results

The literature search retrieved 84 references (Figure 1). We selected the case control studies and summarized them in Table 1.

## 4. Discussions

### 4.1. The Role of INSL3 in the Mechanism of Undescended Testis

INSLR3 is a small peptide (6000 Dalton) with the insulin-typical A-B heterodimeric structure, held together by three internal cysteine bonds. During the intrauterine life, the testis migrates from the abdomen into the scrotum by the 15th to the 28th week of gestation [15]. The first phase (transabdominal) is complete by week 15 of gestation and the second one (inguinoscrotal) by week 35 [1]. The transabdominal phase also implies the gubernaculum expand (bulb swelling, cord shortening), which is controlled by INSLR3, secreted by the Leydig cells. The G178A polymorphic variant of INSL3 can be connected to undescended testes. In the second phase, the testes and its annexes (for example, the epididymis) migrate through the inguinal canal into the scrotum [3,4]. The androgens together with the insulin family of growth factors play an important role in this inguinoscrotal phase [9], being further involved in the actual development and function of the testis as well as the control of growth, metabolism, and reproduction. An undescended testis generally appears due to deviations in the inguinoscrotal phase of testicular formation and descent more than in the transabdominal phase [9].

INSL3 and androgens represent the primary factors involved in gubernacular development during the testicular descent. INSL3 activation is triggered by Leydig cells throughout embryogenesis. It circulates and connects to receptors located on gubernacular cells, leading to a proliferation of the gubernaculum and modifications in his extracellular matrix. This process will conduct to the retraction of the gubernaculum, followed by the migration of the primitive testis from its retroperitoneal position in the scrotum. The main role of INSL3 is to produce a thickening of the gubernaculum, which keeps the testes in the inguinal region and conducts it in the scrotal pouches. It is thereby considered as the first promoter of the transabdominal phase of testicular descent, being a secretory product of Leydig cells, expressed in a differentiation-dependent manner. Anand-Ivell et al. [10] have found measurable levels of INSL3 in the amniotic fluid of the human male fetus from 13 to 16 weeks of gestation, having an exclusively testicular origin and being absent in female fetuses. The result from this study also suggests that INSL3 plays a significant role in the gubernacular swelling reaction, essential for the transabdominal relocation of testes in the first stage of testicular descent. INSL 3 expression in the adult testes was subsequently confirmed using immunohistochemistry and immunoassay. It has been identified as an identical phenotype of bilateral undescended testes in a mutant mouse that presented a mutation of the receptor (RXFP2, Relaxin-like Family Peptide receptor 2) for INSL3 as well, expressed by the mesenchymal cells of the gubernacular ligament. In the human fetus, the synthesis of INSL3 begins early on, following gonadal sex differentiation, at 7 weeks of gestation. INSL3 also acts in the manner of a significant biomarker of the Leydig cell differentiations in the male fetus [6,10].

### 4.2. Expression and Role of INSL3 in Animal Models

As has been previously mentioned, INSL3 plays a key part in the process of testicular formation and descent, mostly in the bulge reaction of the testis gubernaculum [16]. The examination of INSL3 in mutant mice revealed a thin and elongated structure of the gubernaculum in day 16 of gestational age and at birth [16,17]. Between embryonic days 15.5–17.5, the gubernaculum starts developing, a process defined as mesenchymal cell proliferation and differentiation of the outer layers into myoblasts [18]. It is suggested that INSL3 targets the gubernaculum in the male’s mesenchymal core. In this process, androgens may enhance gubernaculum cell proliferation [19]. However, the INSL3-mediated activity excludes a crucial role of androgen in the gubernacular development [20].

**Figure 2:**
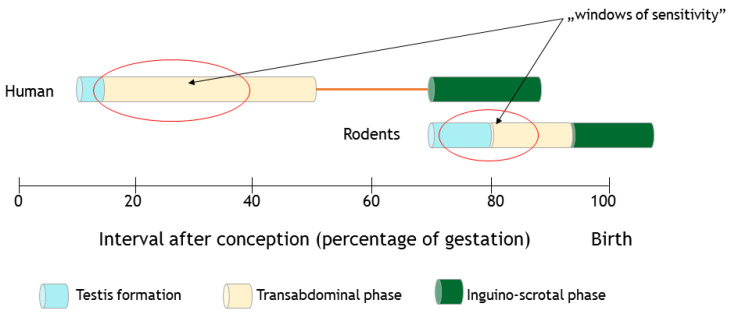
Schematic representation of the timing of masculine gonads formation. The transabdominal and inghino-scrotal phases of testicular descent in rodents and human are represented. Moreover, the estimated ‘windows of sensitivity’ toward endocrine disruptors affecting the male reproductive system are shown in red ovals.

Currently, the data on the physiological functions of INSL3 in rodent studies are related to reproduction and internal fertilization and viviparity [21]. INSL3 transcripts are expressed in the growing gonad and detectable within a time-span of 13.5 days in rats, and starting within 12.5 days of fetal life in mice (Figure 2). In addition, serum levels increase during spermatogenesis in adult males, while in females this is first seen in the ovaries on day 6 after birth [22,23].

Mapping the evolution of INSL3 expression (secretion) into peripheral blood in mice and newborn rats, Anand-Ivell et al. [10] showed that the INSL3 peak is measured in postnatal day 40 in rats and decreases extremely, with a stable sum around postnatal day 60. Elderly rats (around 24 months) have significantly lower circulating INSL3 levels, which is the same as humans. Leydig cell ablation has clarified the process of testis formation and activity. Leydig cell ablation started when Song et al. noticed the consequences of administration of ethane dimethane sulphonate (EDS) in rats [24]. EDS can be used for inhibiting Leydig cells and, thus, for treating and/or preventing Leydig cell dependent diseases. When low-dosage EDS is injected into the testicular arteries, a minimal concentration of testosterone can be achieved and kept below a castrating level. The EDS therapy in young adult rats resulted in no detection of INSL3 in peripheral blood and the loss of a large number of mature Leydig cells and their function. INSL3 was first identified on day 27 after EDS treatment, returning to near normal levels by day 37 [24]. Analyzing the different compartments of peritesticular liquid, the authors noted an elevated concentration of INSL3 in the interstitial fluid, demonstrating that INSL3 crosses the barrier between blood and testis to pass the seminiferous ducts, rete testis, and epididymis to reach an optimal concentration for the specific receptors. These receptors are present on post-meiotic germ cells and also in the head of the epididymis.

In animals with INSL3 defects, compromised testicular descent is the only manifestation [21,25]. The generation of mutant mice for *Insl3* resulted in viable organisms, but presented a clinical bilateral cryptorchid phenotype due to gubernaculum deficiencies, leading, thus, to defective spermatogenesis and later infertility [18,22,26].

In order to verify whether in vivo INSL3-mediated gubernaculum expansion is androgen independent, Adham et al. [20] designed transgenic female and male mice that overexpressed INSL3 in the pancreas throughout fetal and postnatal development. In the INSL3 mutant mice, the expression of transgenic allele extricates the cryptorchidism in the deficient male, suggesting that pancreatic β-cells properly relocated the INSL3 gene outcome to the functional hormone. The same results were reported by Koskimies et al. [27], highlighting the fact that, as adults, no macroscopic differences were found in the inguinal region between INSL3 and wild-type males.

Studies carried out by Tomiyama et al., [15] with the aim of analyzing in detail the testicular descent process and gubernacular enlargement in mice, a transgene insertional mutation found in chromosome 5 to evaluate the role of the G protein-coupled receptor gene, *Great*, revealed that the disarranging of the Great gene leads to failure of the first stage of testicular descent, the transabdominal one [27]. This was identical to that observed in the INSL3-deficient mice. At the 30-day life age, no differences were found in the gubernaculum between mutant males and females, which suggested that *INSL3/Great* signaling regulates gubernacular development [22].

Estrogens have been shown to down regulate INSL3 and thereby cause maldescent of the testis. Investigating whether prenatal exposure to diethylstilbestrol, a synthetic estrogen that can conduct to cryptorchidism in humans and rodents, could interfere with testicular INSL3 mRNA expression, Emmen et al. [18] pointed out that this therapy resulted in a three-fold decrease in the INSL3 mRNA expression level and inhibits the development of the gubernaculum during the transabdominal phase of testis descent.

The occurrence and development of undescended testes in mice is proven to be closely related to exposure to different xenobiotics, which act as endocrine disruptors. The environmental endocrine disruptors are synthetic chemical pollutants, pesticides, and additives that affect the endocrine system. Among them, diethylhexyl phthalate (DEHP) [24] is an organic compound used in plastic products, and a well-known air and food pollutant with endocrine disruptor properties, especially on the male reproductive system, acting as an androgen antagonist. Studying the effect of DEHP on INSL3 mRNA expression in the Leydig cells of mouse embryos and newborn mice, Song et al. [24] showed that INSL3 levels are markedly lower after DEHP therapy. Moreover, DEHP also led to harmful morphological changes in primary Leydig cells cultures in neonatal mice testes. All the data pointed out that the downregulation of INSL3 mRNA by DEHP exposure could determine deviations of the gubernacular swelling reaction process. This might be one of the mechanisms of cryptorchidism development.

### 4.3. INSL3 and His Receptor IGF1 in the Cremasteric Muscle Complex

Apart from INSL3, the genitofemoral nerve and hormones (androgens) are involved in the physiopathology of cryptorchidism [8]. Fetal Leydig cells whose development depends on insulin-like growth factor-1 (IGF1) produce hormones such as androgens and also INSL3 [8]. In rodent models, insulin receptor (INSR) and IGFR1 were confirmed to be mandatory to the development process of the testes. The cremasteric muscle complex is affiliated to the evolution of the gubernaculum and with the LCs. Both are innervated by the genitofemoral nerve (GFN). The division of the GFN will delay the process of testicular descent. Özdamar et al. investigated the presence of IGFR1 in the complex of cremaster muscle and its function in a normally descended testis, and also in undescended testis cases in boys. BothINSL3 and testosterone are mandatory for the expansion of the testis and of the gubernaculum. Testosterone vivifies INSL3 expression in the Leydig cell line, and all these suggestions have shown that during the two phases of testicular descent, the transabdominal and the inguinoscrotal, INSL3 and testosterone-receptor interaction is more condensed than believed. The importance of this interaction in human males was first demonstrated in vivo models, such as rats or mice. In the mentioned study, the authors revealed the existence of IGFR1 in the male cremasteric muscle complex. The density of IGFR1 found on the cremasteric complex in cryptorchid patients was inferior than the value of the control group, thus proving the low stimulation of Leydig cells needed to produce INSL3 [8].

### 4.4. Mutations in INSL3 Gene

The Insulin-like3 gene is composed of two exons incorporating an intron, and encrypted in a single copy in the human genome [9]. An interconnection of G178A-INSL3 gene polymorphism with undescended testis development was cited in animal and human studies, such as in the study of Abou El-Ella et al. [9]. Polymorphism is discovered in the C peptide region of the mutant gene, evolving in amino acid transformation, where the alanine changes to threonine. This amino-acid disruption has been proved to interrupt the normal phases of the process of testicular development [5]. In a study conducted in 2020 by the mentioned author, the interrelation between G178A-INSL3 gene heterogeneity and cryptorchidism was evaluated, and whether or not it contributed to clinical aspects, type, and localization of the undescended testis. The paper concentrated on the essential function of INSL3 in detecting the molecular pathogenic mechanism involved in the gubernaculum differentiation and testicular progression. Abou El-Ella et al. [9] set up a study involving 160 children. The patients were split into two congregations: Group (I) contained 80 boys presenting primary non-syndromic cryptorchidism, and Group (II) contained 80 healthy boys with normally descend testes, and both groups were derived from the general pediatric community. A statistically significant difference between the two groups was noted concerning the genotype results, and also in the allele frequencies of G178A_INSL3 gene polymorphism. GG genotype was commonly observed in the control group, and the heterozygous GA and homozygous AA genotypes was found more often in the cryptorchid group in contrast to the control [9].

Multiple mutations have been researched to explain the implication of INSL3 and its specific receptor, RXFP2, in boys with undescended testis. As stated by Nowacka-Woszuk et al. [14], the frequency of INSL3 mutation is 1–2%, and RXFP mutation–2–4%, although numerous polymorphisms have also been described. This observation led to the suggestion that genetic mutations are mostly seen in the bilateral cases of undescended testes. In a study conducted on an Egyptian cohort, a strong association between G178A-INSL3 polymorphism and cryptorchidism could be observed [14]. In an Indian study, four male brothers were diagnosed with bilateral undescended testes and then isolated, and their complete exome sequencing showed a homozygous missense alteration in the RXFP2 gene (heterozygous for both the mother and the father of the patients) [14]. Poor cell surface expression and failure to bind INSL3 or respond to the ligand with cAMP signaling was observed in the functional analysis of the variant protein, concluding that recessive endowment of variants of the RXFP2 gene can lead to genetic familial cryptorchidism. The phenotype of adults with the s INSL3/RXFP2 mutation could be diverse, from bilateral cryptorchidism with the abdominal testis to retarded testicular descent or else retractile testes. If the INSL3/RXFP2 system is involved in the later stages of the testicular descent process, the result is unclear. Anatomical examination of both unilateral and bilateral undescended testes indicates that other tissues or ligaments can be interfered with because of the altered relative timing of their growth trajectories. In a study conducted by van Brakel et al. [13], insulin-like3 proved the decrease in Leydig cell function for both congenital and acquired undescended testes. Moreover, INSL3 mutations are the same in all types of undescended testes. The functioning of Sertoli cells in patients with bilateral cryptorchidism was highly affected compared to unilateral congenital undescended testes and bilateral acquired cryptorchidism because of the anti-Mullerian hormone.

Testosterone levels and INSL3 vary with age. For example, a small but significant level appears during mini-puberty, and markedly decreased levels are noticed in childhood and then increase during puberty, with the highest levels in adults [26]. de Jong et al. [13] showed that INSL3 levels are lower in adult men with testicular damage involving Leydig cells and with hypo-spermatogenesis compared to male adults with normally descended testes. Van Brakel et al. [13] found low INSL3 levels but with no significant differences between different types of undescended testes. In their paper, men presenting testes that descended spontaneously without medication or surgical management had no altered fertility (defined with testicular measuring volumetry, hormone values, and an assay of semen samples) compared to patients who undergo orchiopexy after an applied ‘watch and wait’ protocol in the childhood period [13]. An undescended testis and contralateral testis that were normally descended were atypical in acquired unilateral undescended testes [18]. This proves that all the malfunctions found were a consequence of a developmental disorder of both testes. The mentioned authors supposed that spontaneous descent, with no treatment or surgical treatment, does not change the final result of INSL3 values or AMH levels in a unilateral acquired undescended testis because treatment does not influence the opposite testis [19].

### 4.5. INSL3 in Amniotic Fluid and Umbilical Cord

INSL3 was also measured in human amniotic fluid collected during routine amniocentesis for prenatal genetic tests. Cohen et al. [10] demonstrated that INSL3 is quantifiable only in the case of male fetuses––at 11 weeks of gestation at the earliest, reaching the highest level during weeks 12–16 of fetal life, and becoming undetectable around week 20, reflecting a reduced Leydig cell production at that moment. Secondly, the composition of amniotic fluid mostly reflects the products of the fetal lungs, kidneys, and amniotic membranes.

Harrison et al. [7] hypothesized that INSL3 concentrations in fetal cord blood is elevated compared to the samples from amniotic fluid, since the last one is considered a diluted source of INSL3. The serum concentration of INSL3 during fetal life can be 5–100 times higher than in the amniotic fluid. Nevertheless, the values in the amniotic fluid are 2–4 times higher in contrast to serum levels in prepubertal boys and young adult men [7]. Fénichel et al. demonstrated that INSL3 concentration in newborn males, in the cord blood, is significantly lower in cryptorchid infants versus the control group, implying that fetal Leydig cell dysfunction may contribute to cryptorchidism. However, in the second trimester, amniotic fluid INSL3 does not differ significantly between normal male infants and those born with cryptorchidism or hypospadias [28].

## 5. Conclusions

This review described the role of INSL3 in testicular descent in the fetal period. INSL3 gene polymorphism is considered a predisposing factor for cryptorchidism.

Testicular descent is a complex process, under the influence of many environmental and genetic factors, which involves in the migration of fetal gonads into the scrotum. INSL3 plays a crucial role in the transabdominal migration of the testes. Leydig cells, which are under the control of IGF1, produce both androgens and INSL3. IGFR1 influences the remodeling and development of the cremasteric muscle complex and doubtlessly the testicular descent. Even if the etiology of cryptorchidism in male boys is unknown, it is hypothesized that it is determined by multiple factors, and is most likely due to the altered signaling of INSL3/RXFP2. The phenotypes of males with *INSL3* mutations vary from bilateral undescended testes, unilateral undescended testes, and the failure of the testes to descend normally into the scrotum at birth, but with a spontaneous descent during the first year of life.

A specific association of *INSL3* gene polymorphism with cryptorchidism was incriminated in both animal and human studies. The ablation of the gene encoding either INSL3 or its receptor, RXFP2, leads to bilateral undescended testes, which are caused by a failure of the gubernacular ligament to expand. The highest concentration of INSL3 in fetal life is proven between 15–18 weeks, from the moment that corresponds to the outgrowth of the gubernaculum and the initialization of the first phase of the testicular descent. This supports the crucial role of INSL3 in the process of testicular descent. Even higher fetal INSL3 levels than supposed initially in the amniotic fluid, in gestational weeks 18–20, sustain the theory that INSL3 has a base role in all phases of testicular descent.

Our review provides insights from a relatively small number of studies on the role of INSL3 in the process of testicular descent. This is one of the reasons for the inclusion of animal studies among the original articles that were reviewed. Apart from this, the animal studies were the first to emphasize the role of INSL3 in the process of testicular descent and this mutation in cryptorchid rodents. A better picture of the role of INSL3 in cryptorchidism would involve harvesting small biopsies from the undescended testes, which might raise ethical issues due to the difficulties of obtaining informed consent [29]. In conclusion:

- Cryptorchidism is one of the most frequent congenital malformations of the genitourinary tract, with the complex pathogenesis still unclear, and with long term consequences on the further life of the male adult (infertility, cancer).
- INSL3 is one of the main factors involved in the testicular descent, especially in the gubernaculum development, remodeling, and development of the cremasteric muscle complex.
- INSL3, a protein hormone produced by the Leydig cells also in the fetal life, has a crucial role in the transabdominal phase of the testicular descent process.
- Animal studies were the first to cite INSL3 gene polymorphism as a predisposing factor for cryptorchidism, but the role of INSL3 in human fetal testicular descent and maldescent is less clearly delineated.

## Figures and Tables

**Table 1:** Case-control studies depicting the importance of INSL3 and its receptors in testicular descent in both children and animals.

Authors, Year	Study Sample	Aims	Main Results
Özdamar et al., 2019 [8]	-15 cremasteric muscle complex (CM) samples from 15 patients aged 1–6 years-UDG (undescended group) -15 male patients-9 to 12 years-CG (control group)	-To investigate if insulin-like growth factor receptor-1(IGFR1) is retrieved in the cremaster muscle complex (CM) and the association between testicular descent and INSL3	-IGFR1 density in the CM was significant lower in the UT cases compared to CG -INSL3 and testosterone are required in the rearrangement of the gubernaculum, and with IGFR1 they induce testicular descent, promoting the inguinoscrotal phase
Abou El-Ella et al., 2020 [9]	-160 children: 80 non syndromic UDT (undescended testis) and 80 healthy.	-To investigate the association between G178A/INSL3 polymorphism and cryptorchidism	-Allele of G178A INSL3 variant is significantly found in the UDT (26.2% vs. 12.5% in controls) -G178A—INSL3 gene polymorphism is a predisposing factor for cryptorchidism, and is seen often in cases with a family history -Family history is a significant predictive risk factor for cryptorchidism, especially associated with consanguinity
Anand-Ivell et al., 2018 [10]	-25,105 samples of live-born male (offspring pregnancies) amniotic fluid samples (collected by routine amniocentesis)	-To investigate if cryptorchidism is significantly associated with increased amniotic concentration of INSL3 (13–16 weeks of gestation)	-In the second trimester, concentration of INSL3 in the amniotic fluid is maximal -Cryptorchid cases have significantly higher INSL3 concentration compared to controls
Harrison SM et al., 2019 [7]	-12 human male umbilical cord blood samples -7 human male testes from fetuses in of fetal life	-To measure INSL3 serum levels in fetal umbilical cord and determine its concentrations during fetal life -To measure INSL3 serum levels and mRNA in the fetal testes	-INSL3 serum concentrations during fetal life (between weeks 15 to 20) were discovered to be 2 to 4 times higher than published prepubertal male levels -Testicular fetal INSL3 mRNA relative expression is proven to be decreased in weeks 14 to 16, rose significantly between 17 and 18 weeks, and returned to decreased values in the 21th week
Yuan et al., 2020 [11]	-Animal research: 6 Ziwuling black goats: 3 pairs with normal and cryptorchid testes—extracted at the age of 6 month (by orchidectomy).	-To investigate anatomical alterations in cryptorchism and to study expression and distribution of INSL-3 in descended/cryptorchid testicular tissues	-A decreased level expression of INSL-3 is remarked in cryptorchid goats compared to controls -The presence of undescended testes caused an important reduction in the spermatogenic epithelium and tubule section
Sinopidis et al., 2019 [12]	-Blood samples -46 male patients-non-syndromic cryptorchidism -43 age-matched controls. =Data grouped according to testicular location.	-To assess the existence of INSL3 allelic variations in the physiopathology of cryptorchidism and to estimate potential consequences on fertility	-Seven forms of a single nucleotide (SNVs) were identified -The cumulative percentage of mutations reported in males with an undescended testis, in contrast to control group–1.8% for INSL3 associated with 2.9% for LGR8 and 4.7% for both -The described variations (A24L, V43L and A60T, and I604V) are discovered in patients with cryptorchidism and also in controls
van Brakel et al., 2017 [13]	-118 adult human males antecedents of UDT (undescended testis) -CUDT (congenital undescended testis (N = 55, 6 bilateral), -AUDT (acquired undescended testis) N = 63 (15 bilateral)	-To detect differences in both Leydig and Sertoli cell function in congenital UDT and acquired UDT using INSL3 and anti-Müllerian hormone (AMH).	-There were no differences found in the function of Leydig on the basis of INSL3 levels between CUDT group and AUDT group -AMH levels for bilateral CUDT were significantly lower compared to bilateral AUDT (6.4 vs. 13.2) -AMH levels in unilateral CUDT is significantly higher compared to bilateral CUDT (12.1 vs. 6.4)
Nowacka-Woszuk et al., 2020 [14]	-Animal studies: dogs -16 undescended testes -21 tests from healthy controls	-To compare the levels of INSL3 in dogs with unilateral cryptorchidism to normal male dogs	-The study showed a significantly different expression of INSL3 genes between cryptorchid dogs and controls -INSL3 transcript level was significantly elevated in undescended testes -The mRNA level of RXFP2 was significantly lower in the undescended gonads compared to scrotal testes

## Data Availability

No new data were created or analyzed in this study. Data sharing is not applicable to this article.
